# Use of a Triaxial Accelerometer to Measure Changes in Gait Sway and Related Motor Function after Corrective Spinal Fusion Surgery for Adult Spinal Deformity

**DOI:** 10.3390/jcm13071923

**Published:** 2024-03-26

**Authors:** Tomoyoshi Sakaguchi, Naveen Sake, Masato Tanaka, Yoshihiro Fujiwara, Shinya Arataki, Takuya Taoka, Yuya Kodama, Kazuhiko Takamatsu, Yosuke Yasuda, Masami Nakagawa, Kayo Utsunomiya, Hiroki Tomiyama

**Affiliations:** 1 Department of Rehabilitation, Okayama Rosai Hospital, 1-10-25 Chikkomidorimachi, Minami Ward Okayama, Okayama 702-8055, Japan; tomoyoshi0127@gmail.com (T.S.); kazuhikopt0803@gmail.com (K.T.); kyushudanji19861007@gmail.com (Y.Y.); ot2632nakagawa@gmail.com (M.N.); www.kayo68@gmail.com (K.U.); 2 Department of Orthopedic Surgery, Okayama Rosai Hospital, 1-10-25 Chikkomidorimachi, Minami Ward Okayama, Okayama 702-8055, Japan; naveen.sake@gmail.com (N.S.); fujiwarayoshihiro2004@yahoo.co.jp (Y.F.); araoyc@gmail.com (S.A.); taokatakuya@gmail.com (T.T.); ykodama314@gmail.com (Y.K.); 3 Hashimoto Artificial Limb Manufacture Co., Ltd., 32-13 Urayasunishimachi, Minami Ward, Okayama 702-8025, Japan; tomiyama@hashimoto.co.jp

**Keywords:** adult spinal deformity, surgery, rehabilitation, spinal balance, gait sway

## Abstract

**Background**: Adult spinal deformity is a complex condition that causes lower back pain, causing spinal imbalance and discomfort in activities of daily life. After corrective spinal surgery, patients’ gait and balance abilities might not revert to normalcy and they might be at increased risk of falling. Therefore, early evaluation of such a risk is imperative to prevent further complications such as a fall, or even worse, fractures in post-surgery ASD patients. However, there has been no report of an investigation of such early changes in gait sway before and after ASD surgery. This is a prospective to investigate changes in gait sway before and following ASD surgery, using accelerometers, and also to examine motor function related to postoperative gait sway. **Methods**: Twenty patients were included who underwent corrective surgery as treatment for ASD, from October 2019 to January 2023. Measurement parameters included a 10 m walking test and the timed up-and-go test (TUG), gait sway was evaluated using accelerometers (root mean square; RMS), and hip flexion and knee extension muscle strength were tested. RMS included RMS vertical: RMS_V_; RMS anterior posterior: RMS_AP_; RMS medial lateral: RMS_ML._ The radiographic spinopelvic parameters were also evaluated preoperatively and postoperatively. *p* < 0.05 was noted as remarkably significant. **Results**: Preoperative and postoperative RMS_V_ were 1.07 ± 0.6 and 1.31 ± 0.8, respectively (*p* < 0.05). RMS_ML_ significantly decreased from 0.33 ± 0.2 to 0.19 ± 0.1 postoperatively (*p* < 0.01). However, RMS_AP_ did not change postoperatively (0.20 ± 0.2 vs. 0.14 ± 0.1, *p* > 0.05). Patients’ one-month postoperative hip flexor muscle strength became significantly weaker (0.16 ± 0.04 vs. 0.10 ± 0.03 kgf/kg, *p* = 0.002), but TUG was maintained (11.6 ± 4.2 vs. 11.7 s, *p* = 0.305). RMS_V_ was negatively correlated with quadriceps muscle strength and positively with TUG. RMS_AP_ was negatively correlated with quadriceps muscle strength. All spinopelvic parameters became normal range after surgery. **Conclusions**: After corrective spinal fusion for ASD patients, the gait pattern improved significantly. Iliopsoas (hip flexor) and quadriceps femoris (knee extensor) muscles may play important roles for gait anterolateral and vertical swing, respectively.

## 1. Introduction

Adult spinal deformity (ASD) causes lower back pain not only with activity but also with rest. Intermittent claudication and gait disturbance have been associated with spinal deformity [1]. It has been reported that worsening sagittal plane alignment is more detrimental to quality of life for ASD patients compared with coronal plane malalignment [2]. While improving sagittal alignment is important, ASD patients take time to adapt to the new posture after corrective surgery, and their ability to walk and balance are temporarily impaired [3,4]. According to gait analysis, preoperative ASD patients walk in a characteristic crouching posture due to spinal sagittal malalignment [5]. After ASD surgery, patients’ gait and balance ability were comparable with those of the general elderly population [6,7]. Usually, gait analysis is generally performed with 3D motion analyzers [6,8] and force plates [9,10,11]. The advantages of these methods are their high reliability and validity, and they aid in advanced analysis and providing a detailed understanding of functional impairment. The disadvantages of these methods are their cost-effectiveness, the complicated operation of the equipment, and the analysis process.

**Figure 1:**
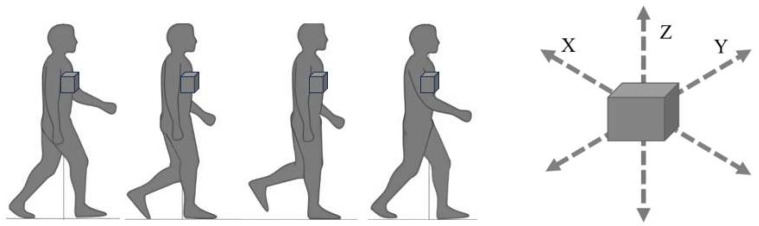
The principle of the accelerometer.

In recent years, gait sway evaluation using accelerometers (wearable sensors) has become a new method of gait evaluation, partially due to good cost-effectiveness [12,13,14]. Accelerometers are easy to wear and have no limitations on measurement location, making them simple and practical tools in clinical practice [15]. The root mean square (RMS) of trunk acceleration has been used as one of the indicators of gait sway, measured using accelerometers [16]. This parameter constitutes a statistical measure of the magnitude of acceleration of X, Y, and Z axes (Figure 1). RMS represents the degree of amplitude of the waveform, and a larger trunk acceleration RMS during gait indicates a greater gait sway. Since postural alignment changes after corrective spinal fusion surgery, walking sway is likely to change.

When ASD patients return to regular life after surgery, their risk of falling may increase without the care of medical staff. Previously, fall risk after ASD surgery has been reported [17]. Therefore, the early evaluation of this risk is very important to prevent fall risk and fractures in ASD patients. Even so, investigations of early changes in gait sway before and after ASD surgery have not been reported. This study aimed to investigate changes in gait sway before and following ASD surgery, using accelerometers, and also to examine motor function related to postoperative gait sway.

## 2. Materials and Methods

**Figure 2:**
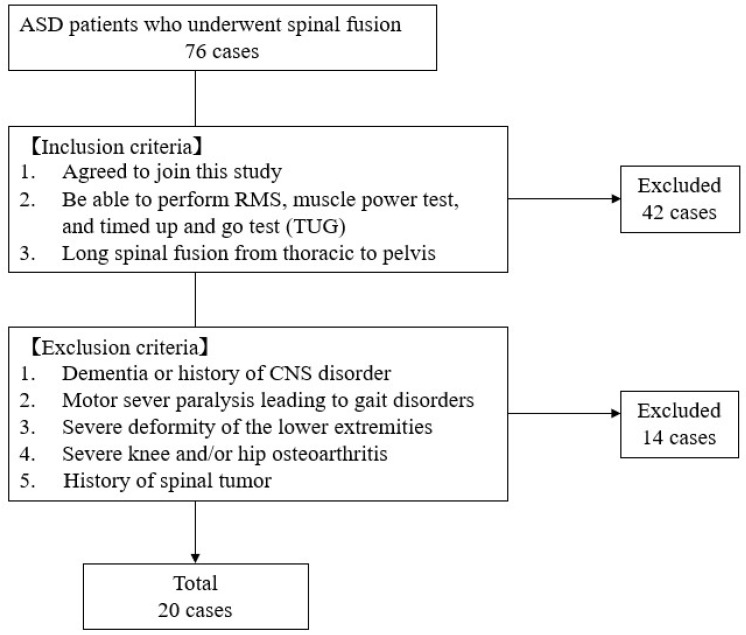
Patient selection.

Between October 2019 and January 2023, 76 patients underwent corrective spinal fusion surgery for ASD at our hospital. Inclusion criteria were as follows: (1) the patients agreed to join this study; (2) the patients were able to perform RMS, muscle power, and timed up-and-go (TUG) tests preoperatively and postoperatively (one month); and (3) the surgery performed was a long spinal fusion from thoracic spine to pelvis. Exclusion criteria were: (1) dementia or history of CNS disorder; (2) severe motor paralysis leading to gait disorders; (3) severe deformity of the lower extremities; (4) severe knee and/or hip osteoarthritis; (5) history of spinal tumor (Figure 2).

**Figure 3:**
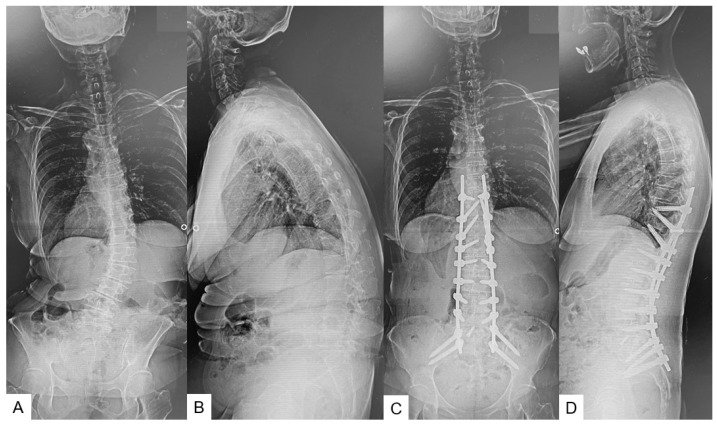
61-year-old female, adult spinal deformity, L1-S1 OLIF and T10-pelvis posterior percutaneous fusion. (**A**): Preoperative posteroanterior radiogram, (**B**): preoperative lateral radiogram, (**C**): postoperative posteroanterior radiogram, (**D**): postoperative lateral radiogram.

The surgical indications for ASD surgery were age > 50 years, sagittal vertical axis (SVA) > 95 mm, and pelvic tilt (PT) > 30° [18]. Corrective spinal fusion was performed with OLIF from L1 to L5 or S1 as a two-stage posterior fusion (Figure 3). In total, twenty patients were included in this study. This timed analysis was acceded to by the review board of our establishment (No. 351).

## 3. Measurement Outcome

### 3.1. Patients Demographic

Patient factors were age at surgery, gender, height, weight, and body mass index (BMI) (Table 1).

Surgery time, blood loss, surgical technique (OPEN, MIS), and upper instrumented vertebra for first-stage oblique lumber interbody fusion (OLIF) and second-stage posterior corrective fusion were investigated as surgery-related factors. (Table 2).

### 3.2. Gait Analysis

Subjects were asked to walk at a comfortable pace on a 10 m walking path. The 10 m walking test has been reported to have excellent reliability [19]. During this analysis, acceleration data for five walking cycles were obtained from a triaxial accelerometer (measurement frequency: 100 Hz) manufactured by Hashimoto’s Prosthetic Limb Manufacturing Co. (Okayama, Japan) The acceleration data were converted to an absolute coordinate system, and the root mean square (RMS) of each directional axis by the square of the walking speed (RMS vertical: RMS_V_; RMS anterior posterior: RMS_AP_; RMS medial lateral: RMS_ML_) was used as an index of walking sway. The formula for calculating RMS is shown below:

$$\mathrm{RMS}\;(m/s^{2})\;=\;\frac{\sqrt{X_{1}+X_{2}+\ldots +X_{n}}}{n}\times \frac{1}{V^{2}}$$

**Figure 4:**
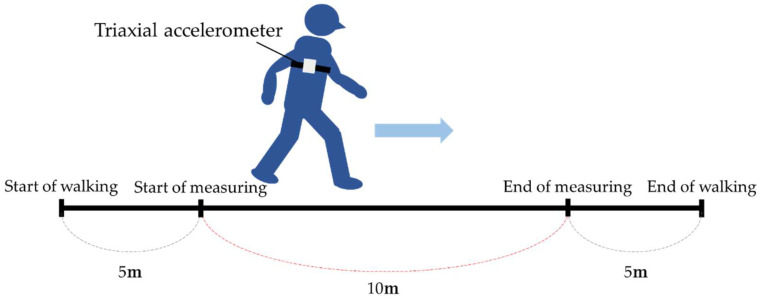
Gait analysis.

**Figure 5:**
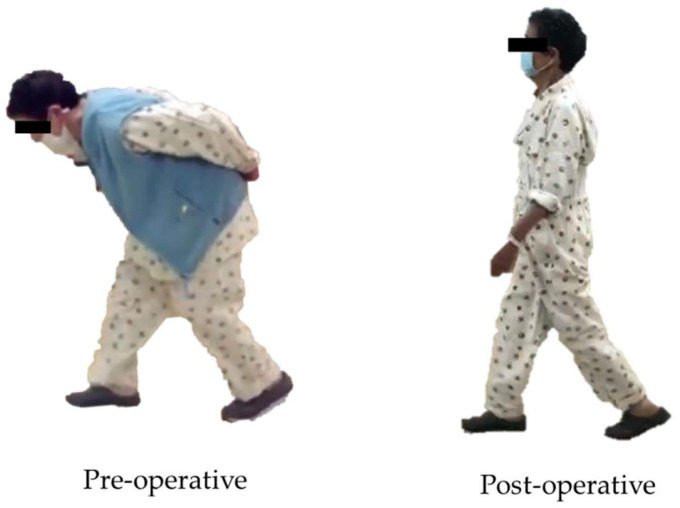
Gait change before and after surgery.

The triaxial accelerometer was attached to the back with a belt and fixed to the back using a xiphoid process as a landmark. Measurements were taken without wearing a trunk corset (Figure 4 and Figure 5).

### 3.3. Muscle Strength

Hip flexion muscle strength (HF) and knee extension muscle strength (KE) were measured. A hand-held dynamometer: HHD (Moby MT-100) manufactured by Sakai Medical (Tokyo, Japan) as used to measure muscle strength. Measurements were taken twice on both sides and the maximum value was normalized by body weight (kgf/kg). Adequate rest was advised between tests to avoid fatigue. The limb position for measuring lower limb muscle strength was based on the previous literature [20].

### 3.4. Timed Up-and-Go Test (TUG)

**Figure 6:**
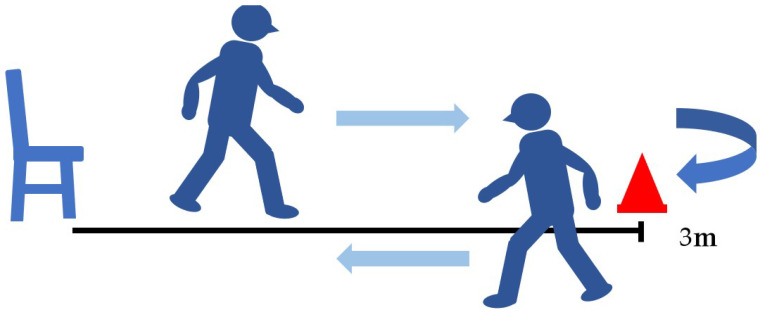
Timed up-and-go test (TUG).

The timed up-and-go test (TUG) records the number of seconds it takes a patient to get up from a chair, walk 3 m around a cone, and return to a chair [21,22] (Figure 6). The TUG has been reported to assess dynamic balance ability and is associated with activities of daily living and falls in the elderly. It has excellent reliability [23].

### 3.5. Radiographic Measurements

**Figure 7:**
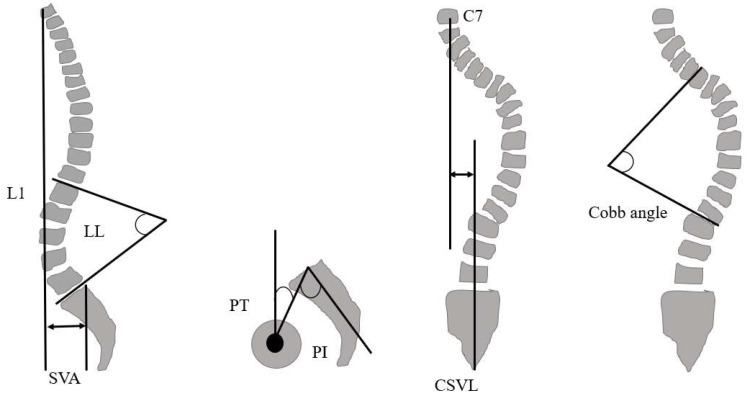
Spinopelvic parameter. SVA, sagittal vertical axis; PI, pelvic incidence; PT, pelvic tilt; LL, lumber lordosis; CSVL, center sacral vertical line.

The radiographic spinopelvic parameters (lumbar lordosis, LL; pelvic tilt, PT; sagittal vertical axis, SVA; pelvic incidence, PI; central sacral vertical line, CSVL; Cobb angle) were evaluated. (Figure 7). Recovery rate (RR) was calculated from the preoperative and postoperative measurements.

## 4. Statistical Analysis

We performed the Shapiro–Wilk normality test and prepared a histogram for the values. The result was not a normal distribution. The one-sample Wilcoxon signed-rank test was used to compare RMS, lower limb muscle strength, TUG, and spinal and pelvic parameters before and after surgery. Spearman’s rank correlation coefficient test was used to correlate postoperative RMS with RR of lower limb muscle strength, TUG, and spinal pelvic parameters. The software utilized to process the data was the EZR (version 1.64) [24] and *p* < 0.05 was noted as remarkably significant. All numerical values were calculated as mean ± standard deviation (SD).

## 5. Results

### 5.1. Comparison of RMS before and after Surgery

**Figure 8:**
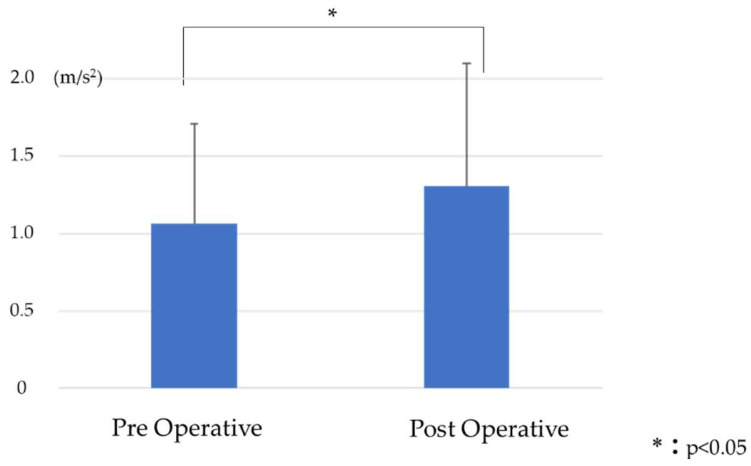
Comparison of RMS_V_ before and after surgery.

**Figure 9:**
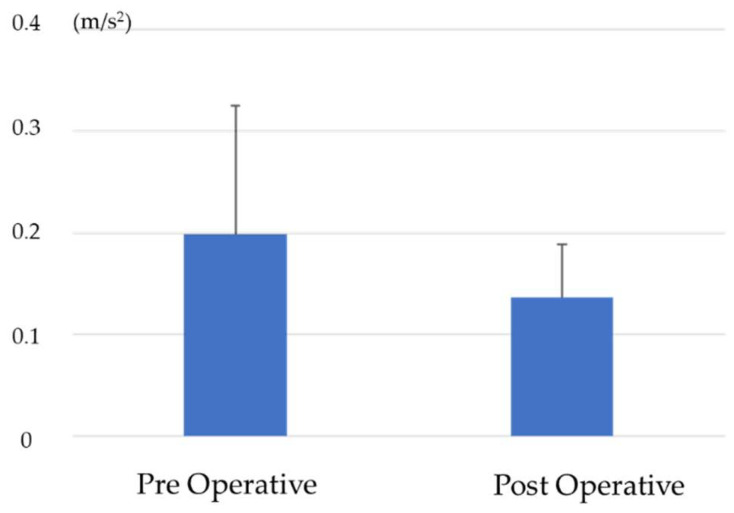
Comparison of RMS_AP_ before and after surgery.

**Figure 10:**
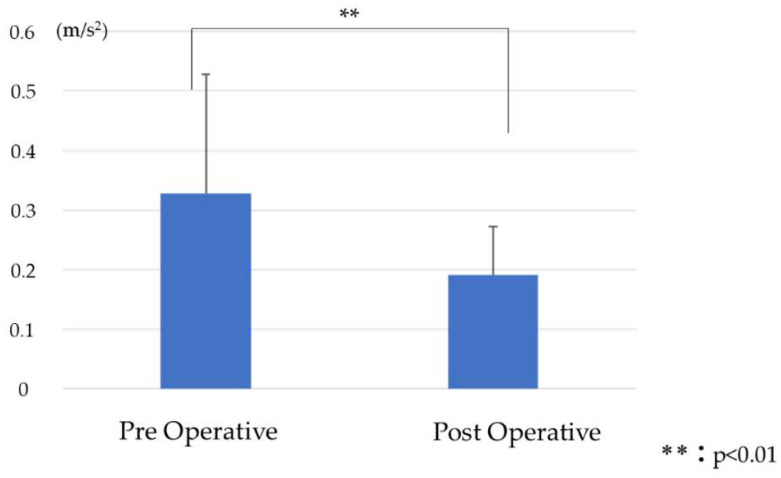
Comparison of RMS_ML_ before and after surgery.

RMS_V_ was 1.07 ± 0.6 preoperative and 1.31 ± 0.8 postoperative, with a significant postoperative increase (Figure 8). RMS_AP_ was 0.2 ± 0.2 preoperative and 0.14 ± 0.1 postoperative, with no significant difference (Figure 9). RMS_ML_ was 0.33 ± 0.2 preoperative and 0.19 ± 0.1 postoperative and significantly decreased postoperatively (Figure 10).

### 5.2. Comparison of Lower Extremity Muscle Strength and Timed Up-and-Go Test before and after Surgery

HF was 0.16 ± 0.04 kgf/kg pre-operative and 0.1 ± 0.03 kgf/kg post-operative and significantly decreased postoperatively. KE was 0.31 ± 0.07 kgf/kg preoperative and 0.28 ± 0.09 kgf/kg postoperative, with no significant difference between pre- and postoperative. TUG was 11.6 ± 4.2 s preoperative and 11.7 ± 2.9 s postoperative, with no significant difference between pre-and postoperative (Table 3).

### 5.3. Comparison of Spinal Pelvic Parameters before and after Surgery

SVA, LL, PT, PI-LL, Cobb, and CSVL showed significant differences between pre-and postoperative, and sagittal and coronal alignments were improved (Table 4).

### 5.4. Correlation between Postoperative RMS and Improvement in Spinal Pelvic Parameters

A correlation was found between postoperative RMS_AP_ and SVA-RR. (*r* = −0.47) (Table 5).

### 5.5. Correlation between Postoperative RMS and Lower Extremity and Timed Up-and-Go Test

A correlation was found between postoperative RMS_V_ and postoperative KE (*r* = −0.625). There was a correlation between postoperative RMS_V_ and postoperative TUG (*r* = 0.77), and a correlation between postoperative RMS_AP_ and postoperative HF (*r* = −0.58) (Table 6).

## 6. Discussion

Adult spinal deformity (ASD) causes lower back pain and spinal imbalance in the elderly population [1]. Surgical correction is necessary to prevent the progression of the deformity and to relieve complaints of pain [25]. However, postoperatively, the elderly find it difficult to adapt to the new posture following surgery for ASD [7]. Regaining spinal balance by physical therapy after surgery is a key to avoiding accidental falls due to spinal imbalance [26]. Furthermore, the risk of a femoral neck fracture due to falls after ASD surgery has been reported [17]. A significant factor among walking and balance tests in post-operative ASD patients was the timed up-and-go test (TUG), which correlated with the Oswestry disability index (ODI) after ASD surgery [27,28]. In recent years, many gait analyses have been performed using accelerometers to calculate RMS, a measure of gait sway in orthopaedic disorders [29,30,31]. However, there have been no reports using accelerometers to investigate changes in RMS before and after ASD corrective surgery. In this study, we examined postoperative changes in RMS and related motor function.

Dubousset reported that an energy-efficient posture is to keep the head above the pelvis [32]. In healthy individuals, the vertical shift of the center of gravity (RMS_V_) is lower in the early phase of stance and higher in the middle phase of stance [33]. Inverse pendulum theory is the basis of energy-efficient gait [34]. In this study, RMS_V_ was significantly increased in the postoperative period. The preoperative gait of ASD patients is compensatory for increased SVA by placing the lower limb joints in flexion, reducing horizontal gaze and the shift of center of gravity during gait in a crouched posture [35,36]. This crouched posture results in a less energy-efficient gait [32]. The improvement of spinal alignment after ASD surgery is thought to improve the compensatory crouching posture, resulting in an energy-efficient gait with improved RMS_V_ (Figure 8). RMS_V_ was moderately negatively correlated with KE in our results (*r* = −0.625). KE is believed to work by absorbing the shock associated with the lowering of the center of gravity during the initial contact with the ground while walking [33]. RMS_V_ increases if the lowering of the center of gravity cannot be controlled [37]. On the other hand, RMS_V_ had a strong positive correlation with TUG, which is an indicator of dynamic balance capacity (r = 0.77). Excessive RMS_V_ is associated with the risk of falling [38].

RMS_ML_ was significantly improved postoperatively. In previous studies, the preoperative gait of ASD patients showed different stride lengths between the left and right sides and uneven floor-reaction forces compared with the general elderly population [7]. Large values of preoperative Cobb angle and CSVL indicate coronal plane malalignment and lateral trunk shift. This might affect stability during the stance phase of gait, altering the stride and worsening RMS_ML_. Although corrective fusion surgery emphasizes improving the sagittal alignment, improvement of coronal alignment also improves RMS_ML_ and stabilizes gait.

**Figure 11:**
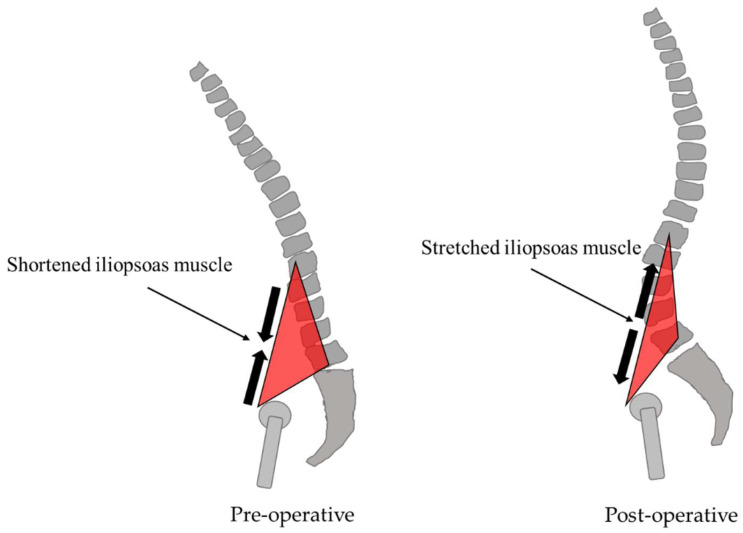
The relationship between iliopsoas muscle stretching.

RMS_AP_ had a moderate negative correlation with postoperative HF and SVA-RR (r = −0.58 and −0.47). The Iliopsoas muscle (IPM), a representative muscle of HF, has an important role in hip flexion as well as spinal column support as a trunk muscle [39] and contributes to maintaining upright posture [40]. Before ASD surgery, the IPM was shortened due to the compensatory crouching posture caused by worsening SVA, and the trunk support function was considered to have been failing. The muscle force of the IPM decreases when the spine is upright and the hip is extended [41]. The shortened IPM is considered to have been stretched after corrective fusion surgery, resulting in decreased muscle force, lower limb swing, and trunk posture retention during gait, and increased RMS_AP_ during gait (Figure 11).

These results suggest that the improvement of muscle power of HF and KE may be a key to reduce RMS after ASD surgery. Previous reports emphasized that pre-surgical physiotherapy increased walking ability and lower extremity strength in patients with degenerative lumbar spine disorders compared with waiting-list controls. [42]. ASD patients usually have muscle weakness before surgery due to less daily activity. Preoperative muscle exercise of lower extremities, especially HF and KE, is recommended. Furthermore, trunk muscle training for seniors was reported to be important because this exercise improves functional mobility (TUG) [43]. With adequate muscle exercise, trunk muscle strength increased by 26% for extension and 23% for flexion at the 12-month postoperative follow-up [44]. Corrective spinal fusion for ASD patients is relatively invasive surgery, and the trunk muscle of the patient may be damaged. Preoperative and postoperative muscle exercise are the keys to important improvements in the activities of daily living for ASD patients.

Limitations of this study included the small sample size and the inclusion of only females. However, our results are generalizable as ASD is more prevalent in females, as previous reported [45]. Joint angles of the lower extremities in the ASD patients were not assessed. It is unclear from the data collected whether improved crouching posture is associated with improved energy expenditure and walking endurance. Furthermore, the long-term course of the study is unknown, as it is a comparison of preoperative and one-month postoperative results. Therefore, it is necessary to increase the sample size and determine the long-term extent to which improvement in crouch walking in ASD improves energy expenditure, walking endurance, and quality of life.

## 7. Conclusions

After corrective spinal fusion for ASD patients, RMS_V_ increased, RMS_ML_ improved, and lateral sway was reduced. RMS_V_ was associated with postoperative muscle strength of the knee extensor and TUG. Therefore, physical therapy programs may need to concentrate attention on prevention of accidental falls and strengthen postoperative IP and KE to control RMS_V_ and RMS_AP_.

## Figures and Tables

**Table 1:** Preoperative characteristics of the study sample.

Patients	n = 20
Age at surgery (year)	71.2 ± 8.9
Sex (male/female)	0/20
Height (cm)	149.1 ± 6.9
Weight (kg)	50.9 ± 9.2
Body Mass Index (kg/m^2^)	22.6 ± 3.7

**Table 2:** Surgery related factors.

**Operation Time (min)**	
1st stage (OLIF)	212.6 ± 48.9
2nd stage (posterior corrective fusion)	270.9 ± 41.8
**Bleeding (mL)**	
OLIF bleeding (mL)	529.2 ± 349.4
Posterior Bleeding (mL)	808.2 ± 441.6
Type of Posterior fusion (OPEN/MIS)	8/12
Upper instrumented vertebra (UIV)	T4:1, T10:19

OLIF: Oblique lumbar interbody fusion.

**Table 3:** Comparison of lower extremity muscle strength and timed up-and-go test before and after surgery.

	Preoperative	Postoperative	*p* Value
HF (Kgf/kg)	0.16 ± 0.04	0.1 ± 0.03	0.002 **
KE (Kgf/kg)	0.31 ± 0.07	0.28 ± 0.09	0.117
TUG (sec)	11.6 ± 4.2	11.7 ± 2.9	0.305

HF, hip flexor muscle; KE, knee extensor muscle; **: *p* < 0.01.

**Table 4:** Comparison of spinal pelvic parameters before and after surgery.

	Preoperative	Postoperative	*p* Value
SVA (mm)	121.8 ± 50.3	42.8 ± 28.7	<0.001 **
LL (degree)	11.7 ± 9.1	42.8 ± 6.3	<0.001 **
PT (degree)	36.2 ± 7.6	25.4 ± 7.4	<0.001 **
PI (degree)	55.7 ± 7.9	55 ± 6.6	0.441
PI-LL (degree)	46.5 ± 11.8	15 ± 8.2	<0.001 **
Cobb (degree)	29.4 ± 25.7	0.53 ± 2.23	<0.001 **
CSVL (mm)	27.5 ± 24.6	6.7 ± 13.2	<0.001 **

SVA, sagittal vertical axis; LL, lumbar lordosis; PT, pelvic tilt; PI, pelvic incidence; CSVL, central sacral vertical line; **: *p* < 0.01.

**Table 5:** Correlation between postoperative RMS and improvement in spinal pelvic parameters.

	SVA-RR	LL-RR	PT-RR	PI-RR	PI-LL-RR	Cobb-RR	CSVL-RR
RMS_V_	−0.13	0.18	−0.28	−0.39	−0.45	0.17	0.17
RMS_AP_	−0.47 *	0.21	0.05	−0.12	−0.18	0.21	0.16
RMS_ML_	−0.06	−0.33	−0.15	0.33	0.41	0.04	0.17

SVA, sagittal vertical axis; LL, lumbar lordosis; PT, pelvic tilt; PI, pelvic incidence; CSVL, central sacral vertical line; RR, recovery rate; *: *p* < 0.05.

**Table 6:** Correlation between postoperative RMS and lower extremity and timed up-and-go test.

	HF	KE	TUG
RMS_V_	−0.19	−0.625 **	0.77 **
RMS_AP_	−0.58 **	−0.08	−0.23
RMS_ML_	−0.37	0.23	−0.33

HF, hip flexor muscle; KE: knee extensor muscle; TUG, timed up-and-go; **: *p* < 0.01.

## Data Availability

The data presented in this study are available in the article.
